# Roles of CUP-5, the *Caenorhabditis elegans *orthologue of human TRPML1, in lysosome and gut granule biogenesis

**DOI:** 10.1186/1471-2121-11-40

**Published:** 2010-06-11

**Authors:** Erin M Campbell, Hanna Fares

**Affiliations:** 1 Department of Molecular and Cellular Biology, Life Sciences South Room 531, University of Arizona, Tucson, AZ 85721, USA

## Abstract

**Background:**

CUP-5 is a Transient Receptor Potential protein in *C. elegans *that is the orthologue of mammalian TRPML1. Loss of TRPML1 results in the lysosomal storage disorder Mucolipidosis type IV. Loss of CUP-5 results in embryonic lethality and the accumulation of enlarged yolk granules in developing intestinal cells. The embryonic lethality of *cup-5 *mutants is rescued by mutations in *mrp-4*, which is required for gut granule differentiation. Gut granules are intestine-specific lysosome-related organelles that accumulate birefringent material. This link between CUP-5 and gut granules led us to determine the roles of CUP-5 in lysosome and gut granule biogenesis in developing intestinal cells.

**Results:**

We show that CUP-5 protein localizes to lysosomes, but not to gut granules, in developing intestinal cells. Loss of CUP-5 results in defects in endo-lysosomal transport in developing intestinal cells of *C. elegans *embryos. This ultimately leads to the appearance of enlarged terminal vacuoles that show defective lysosomal degradation and that have lysosomal and endosomal markers. In contrast, gut granule biogenesis is normal in the absence of CUP-5. Furthermore, loss of CUP-5 does not result in inappropriate fusion or mixing of content between lysosomes and gut granules.

**Conclusions:**

Using an in vivo model of MLIV, we show that there is a defect in lysosomal transport/biogenesis that is earlier than the presumed function of TRPML1 in terminal lysosomes. Our results indicate that CUP-5 is required for the biogenesis of lysosomes but not of gut granules. Thus, cellular phenotypes in Mucolipidosis type IV are likely not due to defects in lysosome-related organelle biogenesis, but due to progressive defects in lysosomal transport that lead to severe lysosomal dysfunction.

## Background

Lysosomes are the major degradative organelles of endocytosed, phagocytosed, and autophagocytosed material [1,2]. Lysosomes also have specialized functions, for example fusing with the plasma membrane to initiate wound repair and mediating some cell death pathways [3-5]. Lysosome biogenesis is a dynamic process, in which late endosomes fuse with lysosomes resulting in a hybrid late endosome/lysosomal organelle [6]. Late endosomes and lysosomes are reformed from these hybrid organelles, a process that requires the release of intra-organellar Ca^2+ ^[6,7].

Some tissues have additional organelles called lysosome-related organelles (LROs) that are acidic, contain some lysosomal proteins, and have cell type-specific functions [8]. Although LROs derive from the endosomal system, they are different from bona fide lysosomes in composition, morphology, and function. Examples of LROs include organelles with storage or secretion functions, such as melanosomes in melanocytes, platelet-dense granules in platelets, and acrosomes in sperm cells [8-10]. In *C. elegans *embryos, gut granules are LROs found in intestinal cells at all stages of development [11-14]. These gut granules contain lipids, birefringent material that is autofluorescent under several wavelengths of light, and gut granule-specific proteins. The function and biogenesis of gut granules is not completely understood.

In *C. elegans*, CUP-5 is required for the biogenesis of lysosomes in scavenger cells called coelomocytes [15,16]. CUP-5 is the sole orthologue of mammalian TRPML1 that is encoded by *MCOLN1*, mutations in which cause Mucolipidosis type IV (MLIV) in humans [15,17]. Many MLIV-associated defects that are linked to lysosomal dysfunction have been described. Some examples include an enlargement of lysosomes that accumulate both lipid and water soluble material, a delay in the transport of endocytosed lactosylceramide (LacCer) from late endosomes/lysosomes to the Golgi Apparatus, a delay in the degradation in and/or transport of endocytosed lipids and proteins to lysosomes, and a delay in the degradation of autophagosome material [18]. Similar to the MLIV phenotypes, worms with a *cup-5 *mutation have enlarged endo-lysosomal compartments in several cell types, including developing intestinal cells and coelomocytes [15,19]. Pulse-chase studies in coelomocytes have shown that CUP-5 is required for the biogenesis of lysosomes, the earliest MLIV-associated defect in the endocytic pathway that has yet been described [16].

Loss of CUP-5 results in embryonic lethality [20]. In these *cup-5 *mutant embryos, there is a significant enlargement of yolk granules and a defect in the degradation of endocytosed yolk proteins in developing intestinal cells [20]. Loss of MRP-4 was shown to rescue both the yolk granule/yolk degradation defects and the embryonic lethality of *cup-5 *mutant [19]. However, MRP-4 is an ATP-Binding Cassette (ABC) transporter that is required for gut granule differentiation in developing intestinal cells [21]. Because of the role of MRP-4 in LRO/gut granule differentiation and the *mrp-4 *mutant rescue of *cup-5 *mutants, we hypothesized that CUP-5 may play a role in LRO biogenesis or differentiation, in addition to its role in lysosome biogenesis. We therefore initiated this study to decipher possible roles of CUP-5 in lysosome and gut granule biogenesis in developing intestinal cells.

## Results and discussion

### Discrete late endocytic organelles and gut granules in the developing intestine

Two late-stage endo-lysosomal organelles (yolk granules and lysosomes) and one LRO (gut granules) have been identified in developing intestinal cells of embryos [11,22]. However, because there are no systematic studies that visualized all three organelles in developing intestinal cells, it is not clear whether they indeed represent discrete and separate compartments. We therefore first carried out colocalization studies in wild type embryos to define these organelles using established markers. We confined our analysis to specific stages of embryonic development, "comma" to "1.5-fold" stages, when the yolk granule defect is clearly observed in *cup-5 *mutants [20].

**Figure 1:**
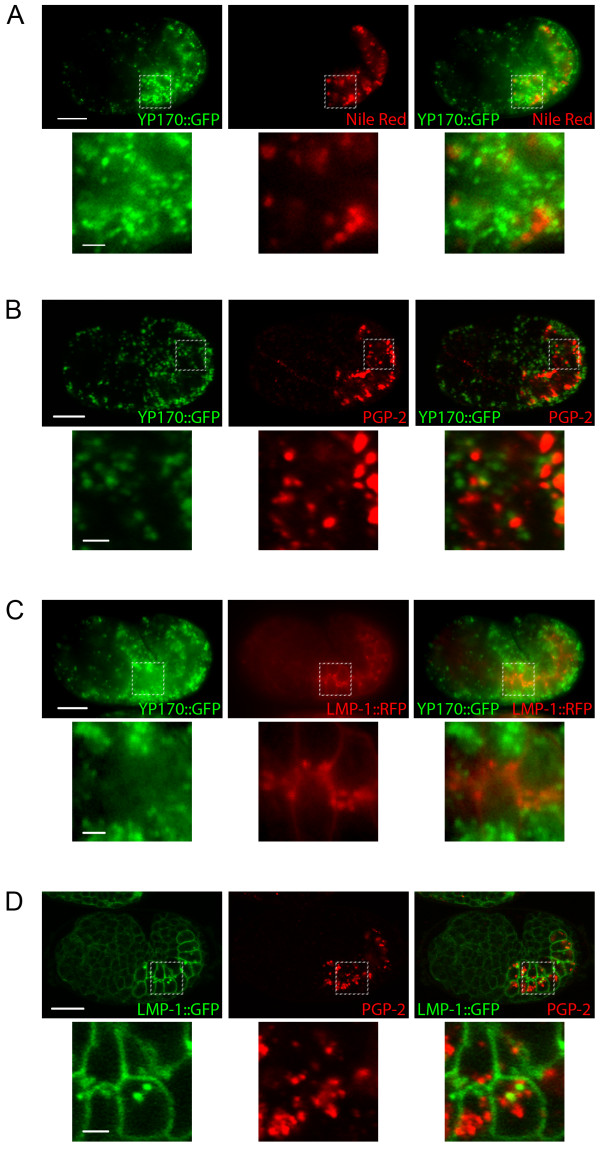
**Late endocytic and lysosome-related organelles in developing intestinal cells of wild type embryos**. A) Epifluorescence images of a wild type embryo that expresses YP170::GFP and stained with Nile Red. B) Confocal images of a wild type embryo that expresses YP170::GFP and immunostained to detect PGP-2. C) Epifluorescence images of a wild type embryo that expresses YP170::GFP and LMP-1::TagRFP(S158T). D) Confocal images of a wild type embryo that expresses YP170::GFP and immunostained to detect PGP-2. Bottom panels are magnified images of the regions indicated in the top panels. Scale bars in whole embryo images represent 10 μm; scale bars in magnified images represent 2 μm.

Yolk granules in developing intestinal cells are late endosomes that contain endocytosed yolk. During oogenesis, yolk is first endocytosed by oocytes. During early morphogenesis, yolk is secreted by all cells into the perivitelline space and is subsequently endocytosed by developing intestinal cells [23,24]. We visualized these yolk granules using transgenic worms that express a fusion of the yolk protein YP170 to GFP (YP170::GFP) [25] (Fig. 1A-C; Additional file 1 Fig. S1B). Lysosomes are terminal compartments that we visualize using an LMP-1::GFP protein reporter (Kostich et al., 2000) (Fig. 1D; Additional file 1 Fig. S1C). LMP-1 protein is the *C. elegans *homologue of mammalian Lysosomal-Associated Membrane Protein 1 (LAMP1) [22]. We also made an LMP-1::TagRFP(S158T) protein reporter, replacing GFP coding sequences with a red fluorescent protein (Fig. 1C). Gut granules are visualized using the lipophilic dye Nile Red staining, UV autofluorescence due to the presence of lipofuscin, or by PGP-2 protein immuno-staining (Fig. 1A, B, D; Additional file 1 Fig. S1) [11,12,19,26]. PGP-2 is an ABC transporter that localizes to gut granules and is required for their biogenesis [26].

Yolk granules, lysosomes, and gut granules represent distinct compartments of developing intestinal cells. YP170::GFP did not colocalize with any of the gut granule markers at steady state (Fig. 1A, B; Additional file 1 Fig. S1B). This indicates that yolk granules are discrete and separate from gut granules. Similarly, YP170::GFP did not colocalize with LMP-1::TagRFP(S158T) at steady state (Fig. 1C). This indicates that yolk granules and lysosomes are distinct compartments; yolk is likely transported to lysosomes but is degraded in these organelles. Finally, LMP-1::GFP did not colocalize with PGP-2 or autofluorescence at steady state (Fig. 1D; Additional file 1 Fig. S1C). This indicates that lysosomes and gut granules are also discrete and separate organelles. Having defined the identities of these organelles by light microscopy studies, we then did localization and mutational analyses to decipher roles of CUP-5 in organelle biogenesis and transport in developing intestinal cells.

### *cup-5(zu223) *lethality is primarily due to defects in the developing intestine

**Figure 2:**
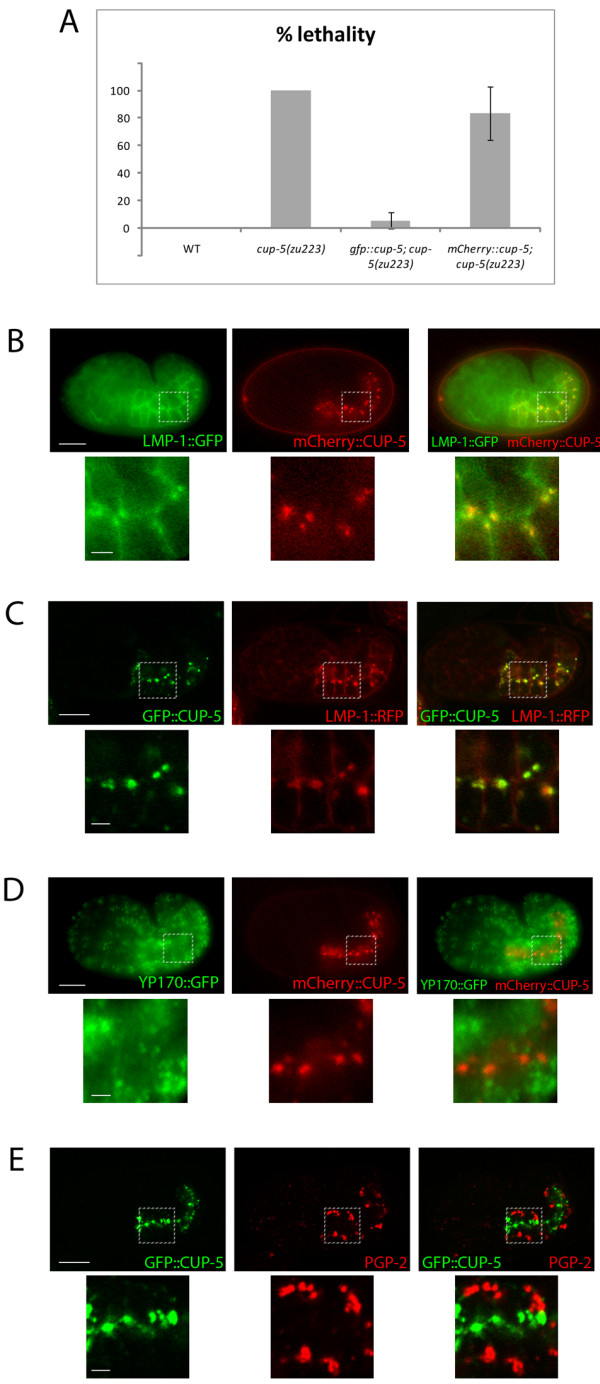
**CUP-5 protein localization in developing intestinal cells**. A) Percent lethality of WT, *cup-5(zu223)*, *cup-5(zu223)*; P*elt-2*::*gfp::cup-*5 and *cup-5(zu223)*; P*elt-2*:: *mCherry::cup-*5 embryos. Error bars represent the 95% confidence intervals for significance. B) Epifluorescence images of a wild type embryo that expresses LMP-1::GFP and mCherry::CUP-5. C) Confocal images of a wild type embryo that expresses GFP::CUP-5 and LMP-1::TagRFP(S158T). D) Epifluorescence images of a wild type embryo that expresses YP170::GFP and mCherry::CUP-5. E) Confocal images of a wild type embryo that expresses GFP::CUP-5 and immunostained to detect PGP-2. Bottom panels are magnified images of the regions indicated in the top panels. Scale bars in whole embryo images represent 10 μm; scale bars in magnified images represent 2 μm.

We had previously made a functional GFP::CUP-5 fusion protein that rescued *cup-*5 mutant lysosomal defects in *C. elegans *coelomocytes [16]. To assay whether this GFP::CUP-5 also rescues intestinal defects in embryos, we expressed this GFP::CUP-5 under the control of an *elt-2 *promoter that drives expression in developing intestinal cells [27]. Although most tissues of *cup-5(zu223) *embryos show lysosomal defects, this P*elt-2::gfp::cup-5 *construct rescues the lethality in *cup-5(zu223) *embryos: 5.4 ± 5.9% lethality compared with 100% without the construct (Fig. 2A). This indicates that the embryonic lethality of *cup-5 *mutant embryos is mostly due to intestinal defects, which is consistent with the strong suppression of *cup-5 *mutant lethality by loss of MRP-4 since *mrp-4 *is only expressed in developing intestinal cells [19,21]. Furthermore, *elt-2 *promoter becomes active after the yolk granule defects manifest themselves in *cup-5 *mutant embryos [20]. This suggests that expression of CUP-5 is sufficient to reverse the endocytic defects of the *cup-5 *mutant, an in vivo model of MLIV.

We also made a P*elt-2::mCherry::cup-5 *expression construct. This mCherry::CUP-5 fusion protein only partially rescues *cup-5(zu223) *embryonic lethality (Fig. 2A), indicating that mCherry interferes with CUP-5 activity. However, mCherry::CUP-5 and GFP::CUP-5 show 100% colocalization (data not shown). In addition, GFP::CUP-5 and mCherry::CUP-5 show the same colocalization with LMP-1 in lysosomes (Fig. 2B, C). Thus mCherry::CUP-5 is a useful marker for the subcellular localization of CUP-5.

### CUP-5 protein localizes to lysosomes

We had previously localized CUP-5 to lysosomes of coelomocytes [16]. We wanted to determine whether CUP-5 localizes only to lysosomes of developing intestinal cells or whether it is also found on yolk granules and gut granules. We used our transgenic worms expressing either GFP::CUP-5 or mCherry::CUP-5 in intestinal cells for these localization studies.

mCherry::CUP-5 fully colocalizes with LMP-1::GFP (Fig. 2B). We see similar colocalization between GFP::CUP-5 and LMP-1::TagRFP(S158T) (Fig. 2C). Note that in addition to lysosomes, LMP-1 also localizes to, or close to, the plasma membrane of developing intestinal cells. Consistent with the lysosomal localization of CUP-5, there was no colocalization between mCherry::CUP-5 and YP170::GFP at steady state (Fig. 2D). We also did not detect any colocalization between GFP::CUP-5 and any gut granule markers (Fig. 2E; Additional file 2 Fig. S2).

Therefore, CUP-5 protein localizes to lysosomes at steady state in developing intestinal cells. We next assayed whether loss of CUP-5 causes content mixing between yolk granules, lysosomes, and gut granules.

### Yolk Granule and lysosome content mixing in the *cup-5(zu223) *mutant

**Figure 3:**
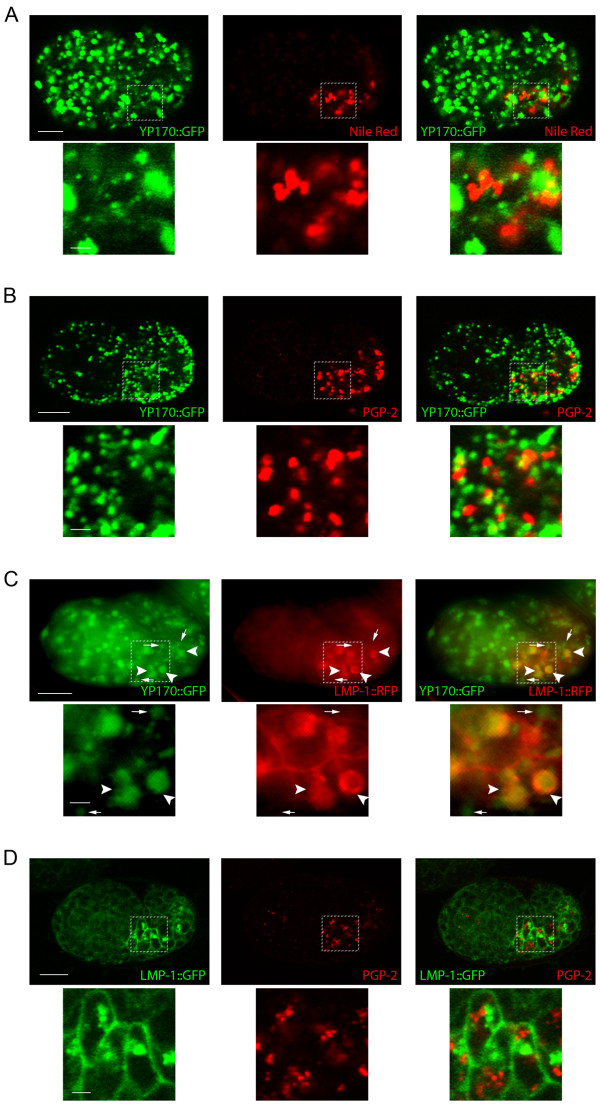
**Late endocytic and lysosome-related organelles in developing intestinal cells of *cup-5 *mutant embryos**. A) Confocal images of a *cup-5(zu223) *embryo that expresses YP170::GFP and stained with Nile Red. B) Confocal images of a *cup-5(zu223) *embryo that expresses YP170::GFP and immunostained to detect PGP-2. C) Epifluorescence images of a *cup-5(zu223) *embryo that expresses YP170::GFP and LMP-1::TagRFP(S158T). Small arrows point to organelles containing only YP170::GFP; large arrowheads point to organelles containing both YP170::GFP and LMP-1::TagRFP(S158T). D) Confocal images of a *cup-5(zu223) *embryo that expresses LMP-1::GFP and immunostained to detect PGP-2. Bottom panels are magnified images of the regions indicated in the top panels. Scale bars in whole embryo images represent 10 μm; scale bars in magnified images represent 2 μm.

To determine whether CUP-5 affects yolk granule, lysosome, and gut granule biogenesis, we did localization studies between markers for yolk granules, lysosomes, and gut granules in *cup-5(zu223) *embryos. Similar to wild type embryos, YP170::GFP did not colocalize with any of the gut granule markers at steady state in *cup-5(zu223) *embryos (Fig. 3A, B; Additional file 3 Fig. S3A). This indicates that yolk granules remain discrete and separate from gut granules in the absence of CUP-5. In contrast to wild type embryos, LMP-1::TagRFP(S158T) localized to the limiting membranes of compartments that contained YP170::GFP in mutant embryos (Fig. 3C). Specifically, we found that all compartments that were labeled with LMP-1::TagRFP(S158T) also contained YP170::GFP (arrowheads in Fig. 3C), but some YP170::GFP-containing compartments did not show detectable LMP-1::TagRFP(S158T) (arrows in Fig. 3C, Additional file 4 Fig. S4). This indicates that there is a transport defect between yolk granules and lysosomes in the absence of CUP-5. Consistent with these results, LMP-1::GFP did not colocalize with the gut granule marker PGP-2 or gut granule lipofuscin (Fig. 3D; Additional file 3 Fig. S3B). Thus in the absence of CUP-5, there is an apparent mixing of content of lysosomes and of yolk granules, while the gut granules remain discrete and separate compartments. This indicates that gut granule biogenesis is normal in the absence of CUP-5. We therefore asked whether there is a defect in the morphologies or content of the various organelles.

### Alteration in size and content of yolk granules/lysosomes in the *cup-5(zu223) *mutant

We had previously shown an enlargement of the total population of yolk granules and an accumulation of YP170::GFP in *C. elegans cup-5(zu223) *mutants [20]. We visualized markers for the different organelles in wild type and *cup-5(zu223) *embryos using identical microscopy parameters that allow us to compare sizes of organelles and intensities of staining of each marker. To determine the sizes of the compartments, we relied on endogenous markers, YP170::GFP for yolk granules, LMP-1::GFP for lysosomes, and autofluorescence for gut granules; this eliminates potential variability due to exogenous staining with dyes. Indeed, Nile Red staining of gut granules showed significant variability in staining quality between different experiments. We also used PGP-2 antibody immunostaining to confirm sizes of gut granules.

**Figure 4:**
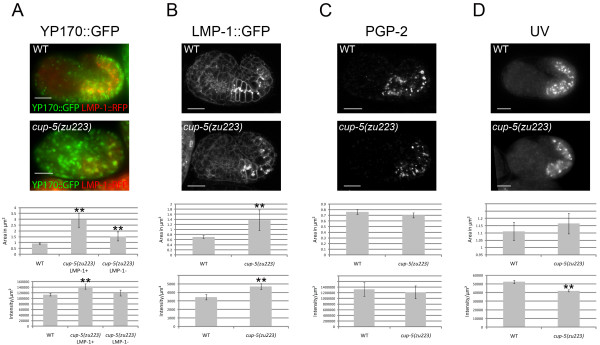
***cup-5 *mutant effects on sizes and intensities of markers of late endocytic and lysosome-related organelles in developing intestinal cells**. Images of wild type and *cup-5(zu223) *embryos, as well as quantification of sizes and intensities per unit area of compartments with YP170::GFP (A), LMP-1::GFP (B), PGP-2 (C), and UV (D) signals. Images for the same marker were taken using the same microscopy parameters. Scale bars represent 10 μm. Error bars represent the 95% confidence intervals for significance, and asterisks represent statistical significance at p < 0.05, compared to WT. In *cup-5(zu223) *embryos, the difference in areas of YP170::GFP organelles (A) with or without LMP-1::TagRFP(S158T) is also statistically significant.

We first confirmed the yolk protein processing defects (Fig. 4A). Because some YP170::GFP-containing compartments contained LMP-1::TagRFP(S158T) and some did not in the *cup-5(zu223) *mutant, we measured the size and signals of both populations of YP170::GFP compartments. These two YP170::GFP-containing populations differed in size and intensity in the *cup-5(zu223) *mutant: compartments that contained only YP170::GFP were significantly smaller than those with both YP170::GFP and LMP-1::TagRFP(S158T) (1.54 ± 0.38 μm^2 ^compared with 2.93 ± 0.61 μm^2^, p-value = 2.44 × 10^-4^) (Fig. 4A). The intensity per unit area of GFP in YP170::GFP-only compartments was also lower than compartments with YP170::GFP and LMP-1::TagRFP(S158T) in the *cup-5 *mutant (p-value = 2.76 × 10^-3^) (Fig. 4A).

We next compared these two populations of YP70::GFP compartments in the *cup-5 *mutant to yolk granules in wild type. The YP170::GFP-only compartments in the *cup-5(zu223) *mutant were larger than YP170::GFP compartments in wild type (1.54 ± 0.38 μm^2 ^compared with 0.92 ± 0.05 μm^2^, p-value = 1.92 × 10^-7^), although the intensities of GFP were similar in both cases (p-value = 0.14) (Fig. 4A). The compartments that contained both YP170::GFP and LMP-1::TagRFP(S158T) in the *cup-5(zu223) *mutant were much larger than YP170::GFP yolk granules in wild type (2.93 ± 0.61 μm^2 ^compared with 0.92 ± 0.05 μm^2^, p-value = 1.80 × 10^-22^), and had a higher intensity of GFP than wild type (p-value = 2.10 × 10^-9^) (Fig. 4A). Thus in the absence of CUP-5, there is a defect in yolk granules/late endosomes prior to the lysosomal expansion described in MLIV cells: yolk granules/late endosomes progressively increase in size before they start accumulating lysosomal markers and showing defects in degradation.

Consistent with the colocalization of LMP-1 and YP170 in the absence of CUP-5, LMP-1::GFP puncta were also larger in *cup-5(zu223) *than in wild type (1.37 ± 0.40 μm^2 ^compared with 0.71 ± 0.05 μm^2^, p-value = 6.19 × 10^-4^) (Fig. 4B). Furthermore, the intensity of LMP-1::GFP was also higher in *cup-5(zu223) *developing intestinal cells, indicating a defect in the turnover of LMP-1 (p-value = 5.02 × 10^-7^) (Fig. 4B).

In contrast to the yolk granule/lysosome defect, there was no apparent effect of the loss of CUP-5 on gut granule size. Gut granule sizes were similar in *cup-5(zu223) *and in wild type embryos based on UV fluorescence (1.17 ± 0.07 μm^2 ^for mutant, 1.11 ± 0.06 μm^2 ^for wild type, p-value = 0.25) and PGP-2 immunofluorescence (0.70 ± 0.04 μm^2 ^for mutant, 0.76 ± 0.04 μm^2 ^for wild type, p-value = 0.06) (Fig. 4C, D). However, the intensity per unit area of autofluorescent material was statistically lower per unit area in *cup-5(u223) *mutant embryos compared with wild type embryos (p-value = 2.3 × 10^-16^) (Fig. 4D), while the intensity per unit area of PGP-2 immunofluorescence was similar in both wild type and mutant embryos (p-value = 0.57) (Fig. 4C). Thus while the biogenesis of gut granules is normal, the loss of CUP-5 may have indirect effects on the composition of these granules; the severe yolk granule/lysosome defect in the *cup-5 *mutant may affect the trafficking of some gut granule proteins.

## Conclusion

**Figure 5:**
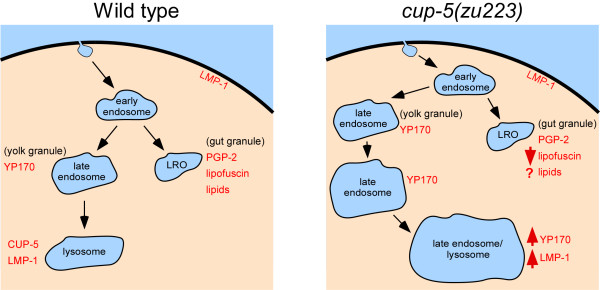
**Model of CUP-5 functions in developing intestinal cells**. Drawings of intestinal cells showing the markers used in this study in wild type and *cup-5 *mutant embryos. Organelles are not drawn to actual scale.

The localization of CUP-5 primarily to lysosomes and the *cup-5 *mutant defects are both consistent with a model where CUP-5 functions primarily in lysosome biogenesis and/or transport but does not function in the biogenesis of gut granules. We propose that YP170 is transported from early endosomes to late endosomes (yolk granules) in both wild type and *cup-5 *mutant embryos (Fig. 5). In wild type, YP170, LMP-1, and CUP-5 are efficiently transported to lysosomes where YP170 is degraded. In the absence of CUP-5, the rate of YP170 and LMP-1 transport to lysosomes is reduced leading to the progressive enlargement of yolk granules (Fig. 5). Eventually, this results in a terminal phenotype, the appearance of large vacuoles that accumulate both YP170 and LMP-1. Gut granules/LROs are presumed to originate from early endosomes. The observation that loss of CUP-5 has a minimal effect on the formation of gut granules indicate that CUP-5 functions downstream of this early endosomal sorting step but upstream of late endosome-to-lysosome transport. However, as this yolk granule/lysosomal defect gets more severe in the absence of CUP-5, it indirectly impacts the trafficking of some gut granule proteins. Our results suggest that MLIV patients will show normal LROs that would not contribute to the symptoms of the disease. Furthermore, our in vivo studies suggest that TRPML1 has functions in late endosome-to-lysosome transport that are earlier than those inferred from some of the terminal lysosomal defects described in cell culture models of MLIV; these terminal lysosomal defects include defective lysosome/autophagosome fusion and reformation and lysosome exocytosis [28-32].

## Methods

### *C. elegans *strains

The following strains were used in this study: GS2886: *unc-36(e251); bIs1(vit-2::gfp; pRF4)*; RT258: *unc-119(ed3); pwIs50(lmp-1::gfp, unc-119(+)*; NP952: *cup-5(zu223) unc-36(e251)/qC1; bIs1(vit-2::gfp; pRF4)*[16,20]. We created, using bombardment methods, strains with the following transgenes [33]: *cdIs184(*P*elt-2::GFP:CUP-5; punc-119(+)-myo-2::GFP); cdIs190(*P*elt-2::mCherry:CUP-5; punc-119(+)-myo-2::GFP)*; *cdIs194(lmp-1::TagRFP(S158T); unc-119(+)-ttx-3::gfp)*. TagRFP(S158T) is a bright monomeric red fluorescent protein [34].

### Plasmid construction

The LMP-1::TagRFP(S158T) plasmid was made by replacing the GFP with TagRFP(S158T) coding sequences in a plasmid that expresses an LMP-1::GFP fusion protein [16]. The ~742 bp TagRFP(S158T) cDNA was PCR amplified (primers CACACAACCGGTAGAAAAAATGGTGTCTAAGGGCGAAGAGC and CACACAGAATTCCAAAGCTTGTGGGCTTTTATTAAGTTTGTGCCCCAGTTTG), restriction digested with *Age*I + *Eco*RI and ligated into the 2979 bp fragment of pHD43 to make plasmid pHD491[35]. The 3532 bp *Sph*I + *Age*I fragment of pHD491 was then ligated to the 3946 bp *Sph*I + *Age*I fragment of pPD117.01-LMP-1 to make plasmid pHD499 used to make transgenic lines.

The GFP::CUP-5 and mCherry::CUP-5 plasmids were made by inserting the *elt-2 *promoter to existing GFP::CUP-5 and mCherry::CUP-5 plasmids (pJF168 and pHD260, respectively). The *elt-2 *promoter was PCR amplified (primers CACACACCGGGCTGCAGGAATTCACATA and CACACACGCGGATCCATTCTATAAT) from pJM260, and restriction digested with *Pst*I and *Bst*UI. Both GFP::CUP-5 and mCherry::CUP-5, pJF168 and pHD260, respectively, were restriction digested with *Bmt*I, incubated with T4 DNA Polymerase to create a blunt end, and then digested with *Pst*I. The promoter fragment (5057 bp) was then ligated to the pJF168 fragment (6074 bp) and pHD260 fragment (6227 bp) to make pHD423 and pHD429, used to make transgenic lines.

### Microscopy

Nile Red is a lipophilic membrane-permeable dye that diffuses across membranes and fluoresces when it complexes with lipids [24,26,36]. For Nile Red staining, adult worms were placed on OP50 bacteria suspended in 60 ug/ml Nile Red, on NGM plates with 60 ng/ml Nile Red (Invitrogen, Carlsbad, CA). Worms were allowed to lay embryos overnight, and embryos were immediately imaged. For imaging of live worm embryos (Nile Red, UV imaging, and fluorescently tagged constructs), embryos were resuspended in M9 buffer and dropped onto 3% agar pads; coverslips were added and sealed with petroleum jelly.

For PGP-2 immunostaining, embryos were dropped onto slides coated with poly-l-lysine (Sigma, St. Louis, MO) and covered with a coverslip. Slides were quickly frozen on a metal plate cooled over dry ice, and coverslips were flicked off the slides. Slides then underwent one 15-minute 100% methanol wash at -20°. Slides were washed twice with PBST (PBS plus 0.1% Tween), and blocked with PBS, 1% nonfat milk, 1% BSA, 0.1% Tween for 2 hours at 4°. Anti-PGP-2 antibody was added at 1:500 dilution in the above blocking buffer and left overnight at 4°, followed by three 10-minute washes in PBST [26]. Secondary anti-rabbit Cy3 antibody (Jackson ImmunoResearch Laboratories, West Grove, PA) was added at 1:200 dilution in blocking buffer for two hours at room temperature, followed by three 10-minute washes in PBST. Excessive moisture was wicked away from embryos, and mounting media (Invitrogen) was added prior to imaging.

Confocal images were taken on a Zeiss LSM510 (Carl Zeiss, Oberkochen, Germany) laser scanning confocal, using LSM imaging software. Epifluorescence images were taken on a Leica DMRXA compound microscope (Leica Microsystems, Wetzlar, Germany), using MetaMorph software (Molecular Devices, Sunnyvale, CA).

For colocalization analysis and quantification of organelle size and intensity, MetaMorph (Molecular Devices) was used, and Microsoft Excel was used for statistical analysis. For each embryo measured, two 7 × 7 μm boxes were drawn, and all compartments within each box were measured for area and intensity per unit area. For YP170::GFP and LMP-1::GFP experiments, one z-section for each of five embryos of each genotype was measured. For PGP-2 and UV experiments one z-section of five embryos of each genotype was measured. We used two-tailed t-test p-values to determine significance in all experiments. Figs. 1, 2, 3 and 4 and Additional files were assembled using Adobe Photoshop (Adobe Systems Incorporated, San Jose, CA), and Fig. 5 was assembled using Canvas (ACD Systems, Victoria, BC Canada).

## Abbreviations

LRO: Lysosome-related organelle; MLIV: Mucolipidosis type IV; ABC: ATP-Binding Cassette.

## Authors' contributions

EMC executed the experiments and wrote the manuscript. HF supervised the studies and edited the manuscript. Both authors read and approved the final manuscript.

## Supplementary Material

Additional file 1 **Fig. S1 - Late endocytic and lysosome-related organelles in developing intestinal cells of wild type embryos**. Supplementary figure showing images of wild type embryos stained to detect various compartments in developing intestinal cells. Click here for file

Additional file 2 **Fig. S2 - CUP-5 protein localization in developing intestinal cells**. Supplementary figure showing images of wild type embryos expressing GFP::CUP-5 and stained to detect gut granules in developing intestinal cells. Click here for file

Additional file 3 **Fig. S3 - Late endocytic and lysosome-related organelles in developing intestinal cells of *cup-5 *mutant embryos**. Supplementary figure showing images of *cup-5 *mutant embryos stained to detect various compartments in developing intestinal cells. Click here for file

Additional file 4 **Fig. S4 - Late endocytic organelles in developing intestinal cells of *cup-5 *mutant embryos**. Supplementary figure showing images of *cup-5 *mutant embryos stained to detect YP170::GFP and LMP-1::RFP in developing intestinal cells. Click here for file
