# Supplementary figures and images for: Roles of CUP-5, the Caenorhabditis elegans orthologue of human TRPML1, in lysosome and gut granule biogenesis
Source: BMC Cell Biol. 2010 Jun 11;11:40. doi: 10.1186/1471-2121-11-40 (PMC2891664; doi:10.1186/1471-2121-11-40)

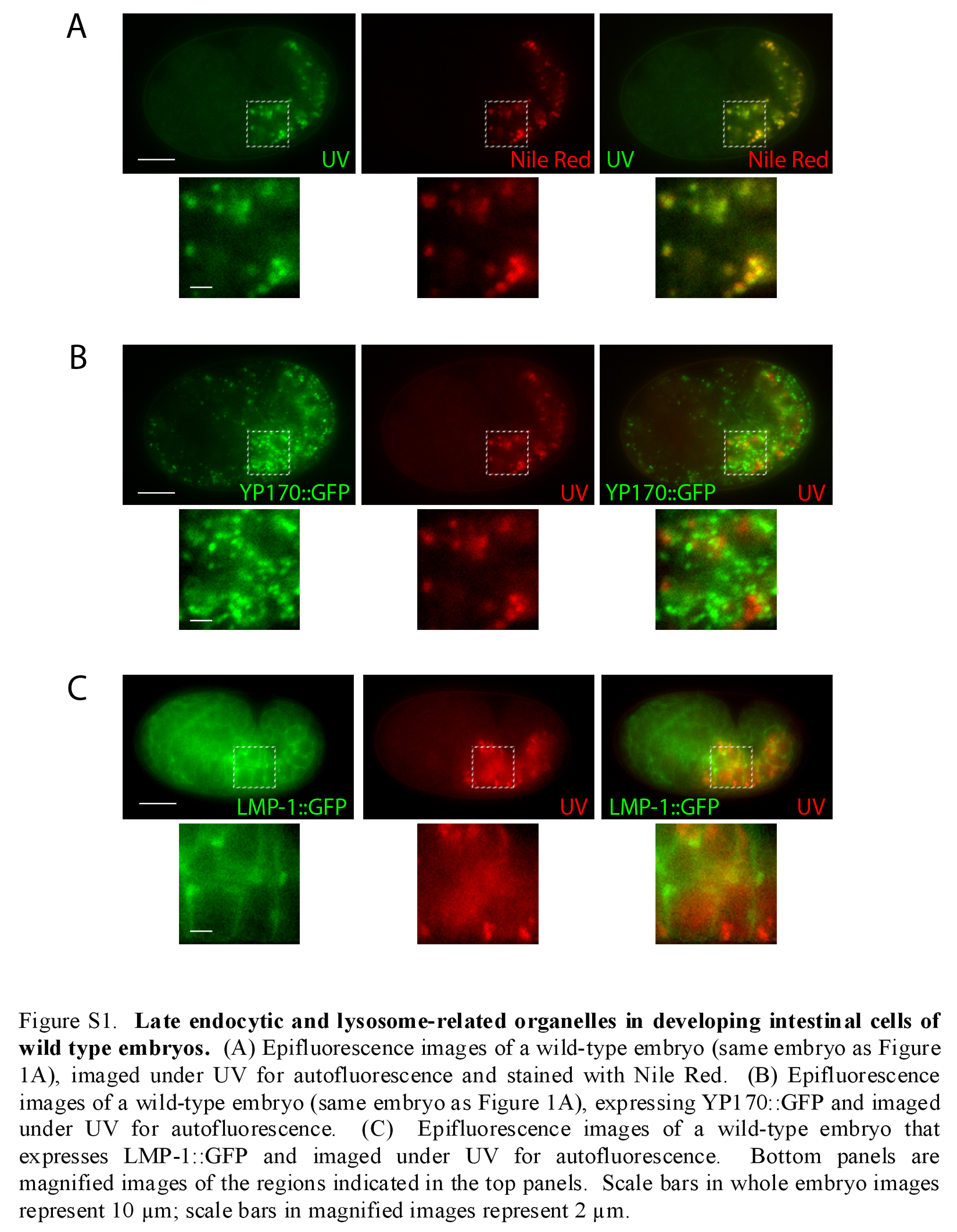

Supplement: Additional file 1 — Fig. S1 - Late endocytic and lysosome-related organelles in developing intestinal cells of wild type embryos. Supplementary figure showing images of wild type embryos stained to detect various compartments in developing intestinal cells. [file 1471-2121-11-40-S1.TIFF]

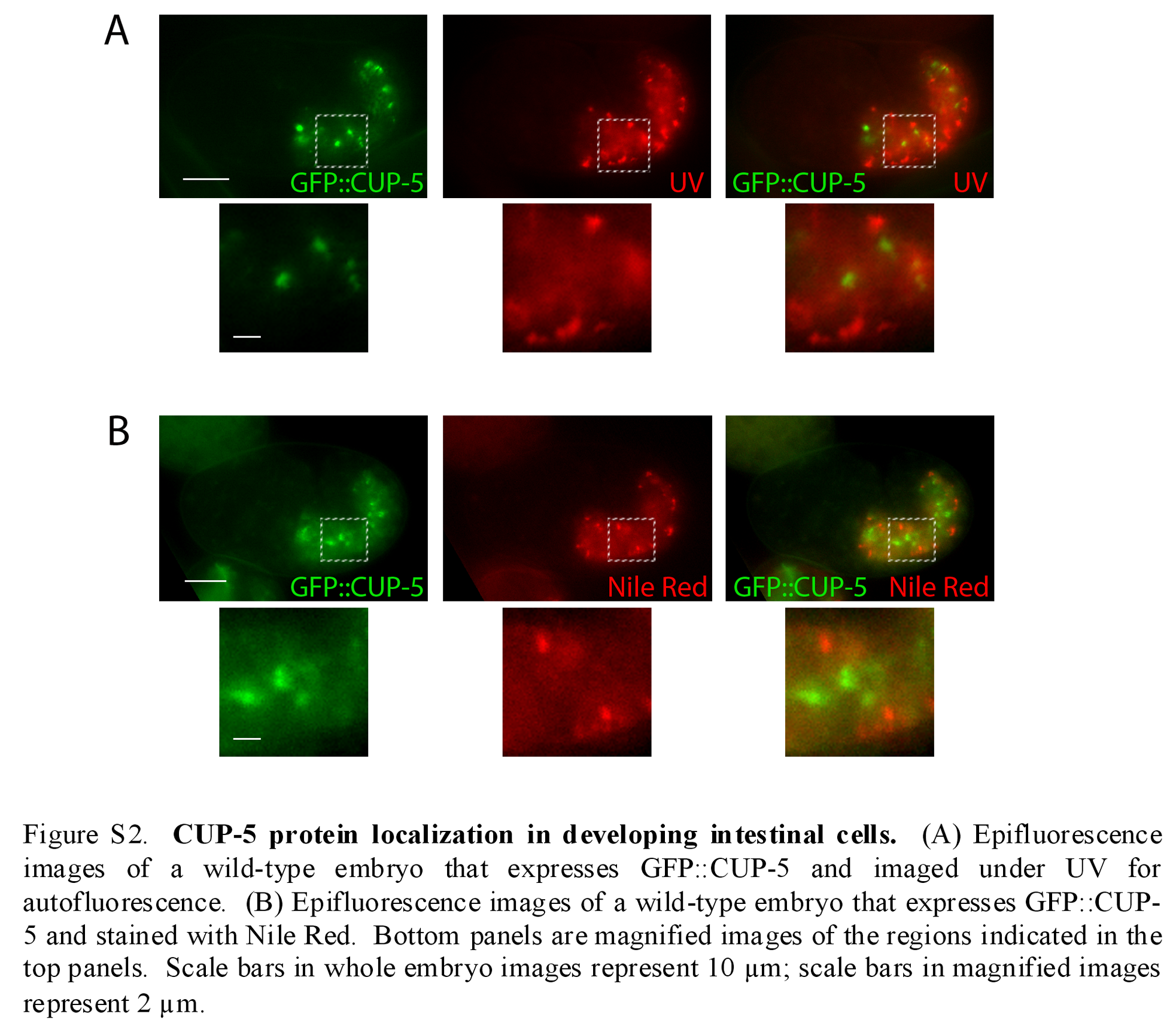

Supplement: Additional file 2 — Fig. S2 - CUP-5 protein localization in developing intestinal cells. Supplementary figure showing images of wild type embryos expressing GFP::CUP-5 and stained to detect gut granules in developing intestinal cells. [file 1471-2121-11-40-S2.TIFF]

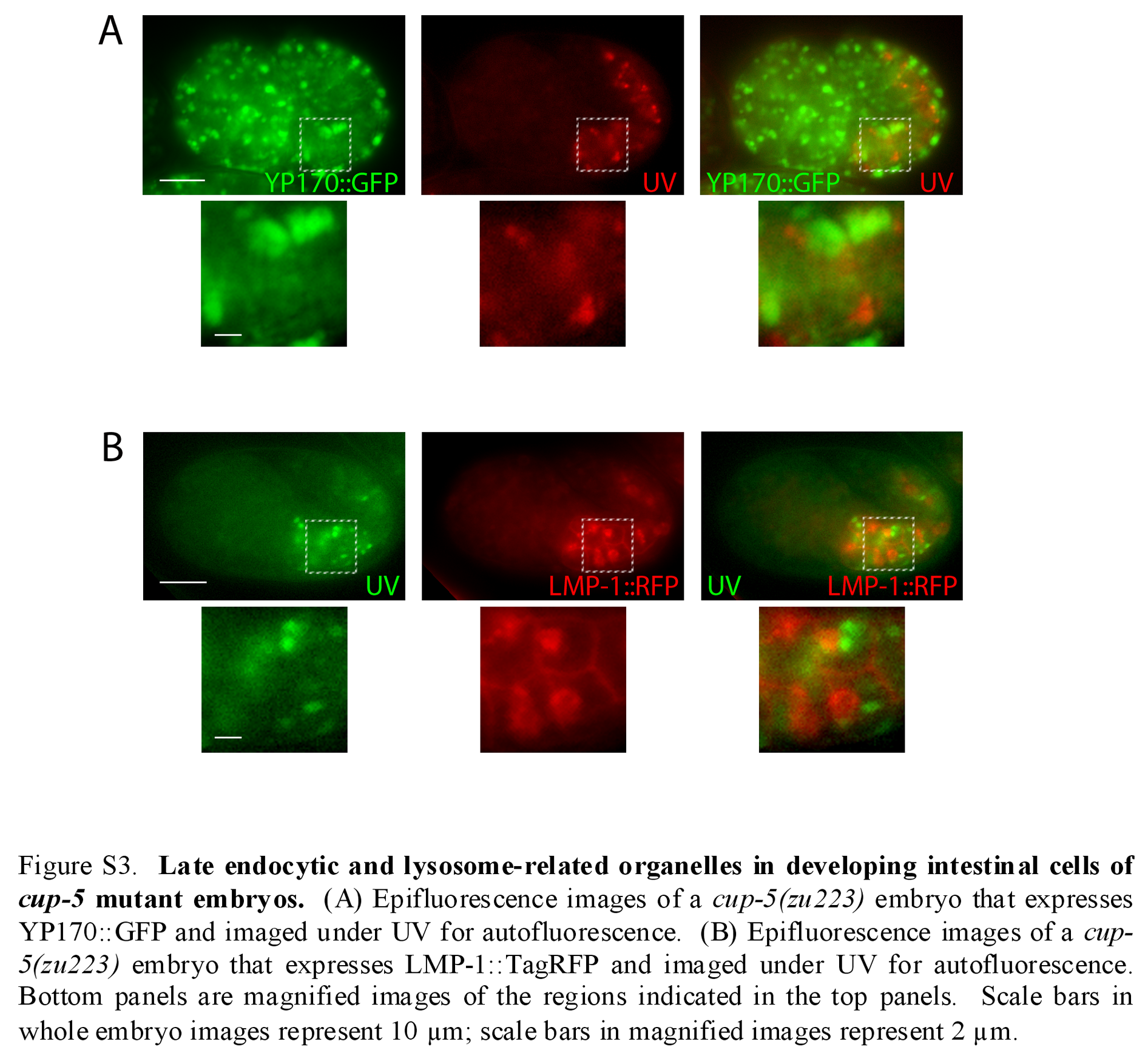

Supplement: Additional file 3 — Fig. S3 - Late endocytic and lysosome-related organelles in developing intestinal cells of cup-5 mutant embryos. Supplementary figure showing images of cup-5 mutant embryos stained to detect various compartments in developing intestinal cells. [file 1471-2121-11-40-S3.TIFF]

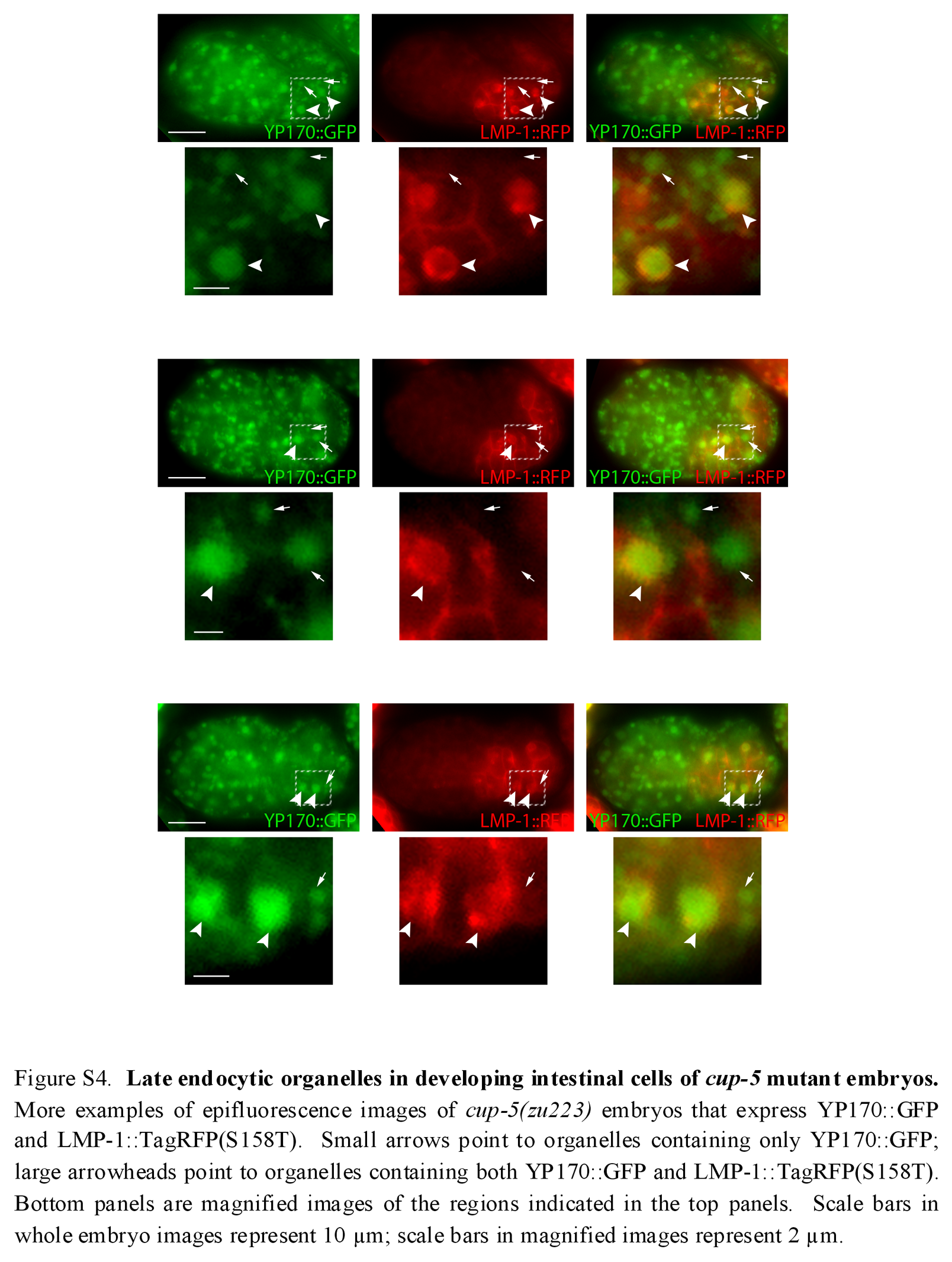

Supplement: Additional file 4 — Fig. S4 - Late endocytic organelles in developing intestinal cells of cup-5 mutant embryos. Supplementary figure showing images of cup-5 mutant embryos stained to detect YP170::GFP and LMP-1::RFP in developing intestinal cells. [file 1471-2121-11-40-S4.TIFF]
